# Automatic detection of mind wandering in a simulated driving task with behavioral measures

**DOI:** 10.1371/journal.pone.0207092

**Published:** 2018-11-12

**Authors:** Yuyu Zhang, Takatsune Kumada

**Affiliations:** Department of Intelligence Science and Technology, Graduate School of Informatics, Kyoto University, Kyoto, Japan; Universite Toulouse III Paul Sabatier, FRANCE

## Abstract

Mind wandering (MW) is extremely common during driving and is often accompanied by performance losses. This study investigated the use of driving behavior measurements to automatically detect mind wandering state in the driving task. In the experiment, participants (N = 40) performed a car-following task in a driving simulator and reported, upon hearing a tone, whether they were experiencing mind wandering or not. Supervised machine learning techniques were applied to classify MW-absent versus MW-present state, using both driver-independent and driver-dependent modeling methods. In the driver-independent modeling, we separately built models for participants with high or low MW and participants with medium MW. The optimal models can not offer a significant improvement than other models. So building effective driver-independent models with the leave-one-participant-out cross-validation method is challenging. In the driver-dependent modeling, we built models for each participant with medium MW. The best models of some participants were effective. The results indicate the development of mind wandering detecting system should take into account both inter-individual and intra-individual difference. This study provides a step toward minimizing the negative impacts of mindless driving and should benefit other fields of psychological research.

## Introduction

Being unintentionally distracted from an intended focus is a common experience of most people. Such distraction could be found in a variety of contexts of daily life [1]. For example, while reading a book, the reader’s attention may drift away from the text towards self-centered matters [2]. After a period of time, the reader may realize that he or she has lost track of reading, indicating the occurrence of mind wandering.

Mind wandering (MW) is a spontaneous, task-unrelated, internal mental process of which the individual is often unaware [3]. It is a form of “looking without seeing” in which the eyes are fixated on an appropriate external stimulus while very little is being processed, as the mind is focused on internal thoughts independent of the stimulus [4]. Individuals differ in susceptibility to mind wandering because of the differences in control capabilities, the number and importance of current life concerns, and the likelihood that those concerns will be triggered by the present context [5, 6].

To know the occurrence of mind wandering, currently there are only two subjective reporting methods. The first is to have participants provide a mind wandering report in response to thought probes placed throughout the task (probe-caught). The second is to allow participants to provide a mind wandering report whenever they catch themselves mind wandering (self-caught). Researchers have linked these mind wandering reports to neurological signals, acoustic and prosodic information, physiological signals, behavioral measures, and eye behaviors [7, 8].

A variety of experimental tasks, including signal detection, reading comprehension, vigilance and memory, have shown that mind wandering is often accompanied by performance impairments [9]. In the present study, we examine mind wandering in the context of driving. It is known that driving is a situation that often induces mind wandering. Drivers who are involved in dramatic personal events or are thinking about personal problems have a higher accident risk [10]. A few studies on mind wandering in driving situations have shown that participants during mind wandering have longer response times to sudden events, drive at higher speeds, maintain a shorter inter-vehicle separation distance [11], and tend to focus visual attention narrowly on the road ahead [9]. Therefore, preventing mind wandering while driving is an important issue for driver safety.

To the best of our knowledge, the present study is the first to attempt to detect drivers’ mind wandering by their driving performance. A few previous studies have detected mind wandering in reading or other tasks, such as [12], [13] and [14]. For example, the participants of Franklin et al. [15] read approximately 5000 words of a novel in a self-paced word-by-word reading task. Mind wandering was detected using a behavioral measure (reaction time). When the text was difficult and a participant responded fast, the participant was predicted to be off task; if a participant responded slowly in a difficult text, he or she was predicted to be on task. Mind wandering could to be classified at a 72% correct rate during reading a difficult text based on reaction time.

A few recent studies have detected mind wandering with machine learning classification methods in a user-independent fashion. In these studies, features were extracted from time series data, such as, behavioral or physiological data, then submitted to classifier to identify mind wandering state. For example, Bixler and D’Mello (2016) [7] automatically detected mind wandering during reading using a computer interface. Extracted features included context features, global gaze features, and local gaze features, which were calculated from windows prior to each mind wandering report. Mind wandering was detected with an accuracy of 72% (kappa = 0.31) when probes were at the end of a page (using a Bayes net algorithm) and an accuracy of 67% (kappa = 0.18) when probes were in the midst of reading a page (using a naive Bayes classifier). Pham and Wang (2015) [16] detected mind wandering using heart rate features calculated from windows before mind wandering reporting and lecture content features, while participants viewed Massive Open Online Courses (MOOCs)-style lectures on a mobile phone. They were able to achieve a kappa value of .22 (accuracy = 71.22%) with a KNN classifier.

In the present study, we detect mind wandering state in a driving task, which is the same task with Zhang and Kumada (2017) [17]. The experiment was conducted in a driving simulator. Participants performed a car following task, requiring them to follow a vehicle and respond to sudden braking of the front vehicle. As a secondary task, they reported their mind wandering state when a tone probe was given. The present approach to mind wandering detection is to estimate mind wandering state by using driving behavior data. We extracted both global and local features from the driving behavior data. With these features, we build models with supervised classification techniques, both driver-independently and driver-dependently, to discriminate instances in which mind wandering subjectively reported present (‘MW-present’) versus absent (‘MW-absent’).

## Experiment

**Fig 1:**
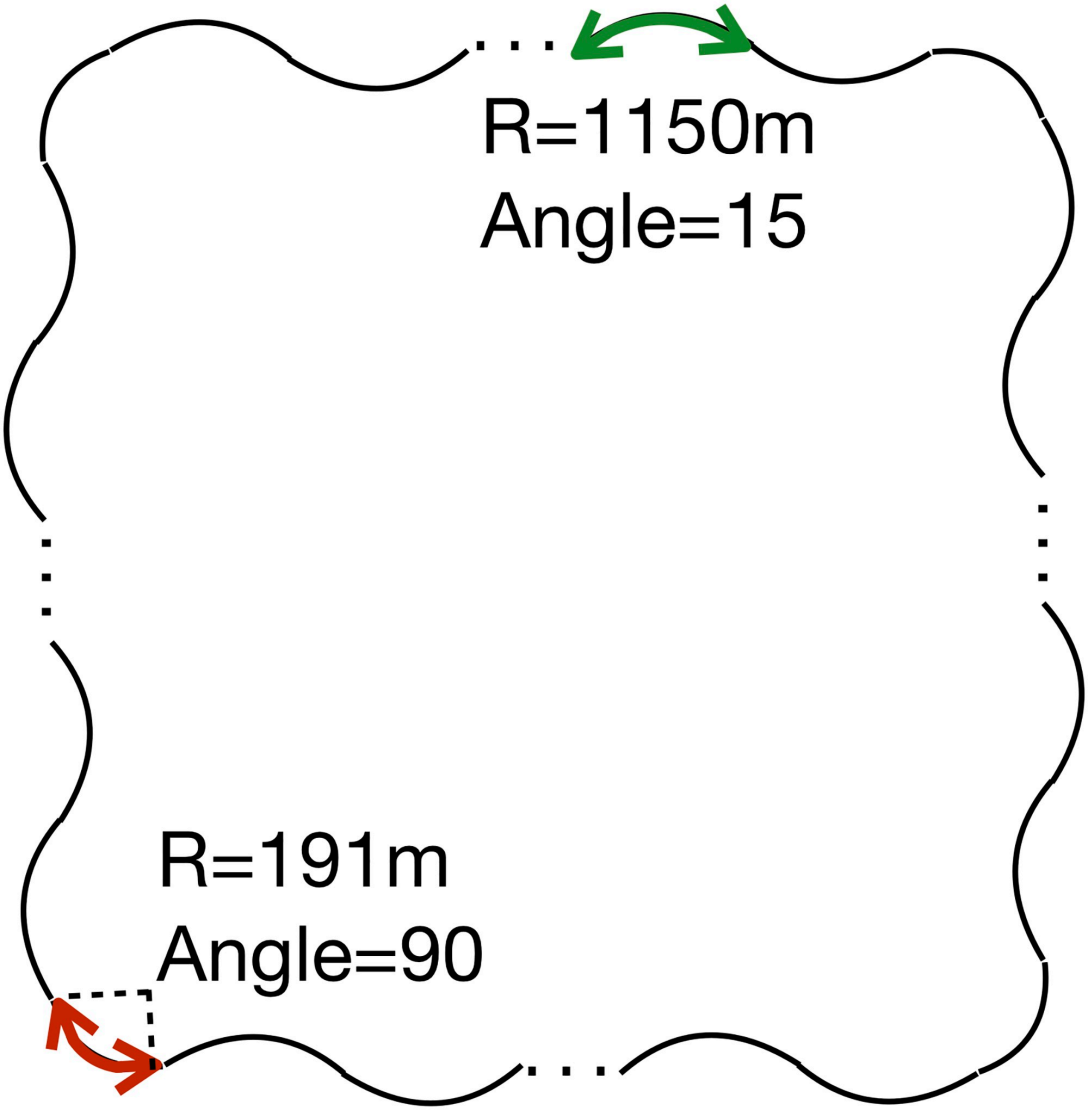
Driving track. The ‘R’ and ‘Angle’ were the radius and central angle of arc. The driving track is similar to square. The edge of driving track was composed of arcs with radius of 1150m and angle of 15. The edges were connected with an arc with radius of 191m and angle of 90.

This study was approved by the Ethics Committee in Unit for Advanced Studies of the Human Mind, Kyoto University. Participants provided their written informed consent to participate in this study. In this experiment, 40 participants, all holding a valid Japanese driver’s license, were recruited from Kyoto University (age range: 20–39 years; mean age: 22.4 years). Data were collected in a driving stimulator, which consisted of a Play-seat Evolution Black + Logitech G27 and a screen (SONY FWD-S42H2, 1920 pixels × 1080 pixels). The driving scene was simulated by UC-win/Road 10.4 and displayed on the screen. The vehicles were driven in good weather and daylight, along the track depicted in Fig 1. The length of the track was 25,270 meters. No other traffic was set on the road except for the participant’s car and the front car.

Before the experiment, participants practiced keeping a 20 m distance from the lead vehicle and became familiar with the present task, car-following with mind wandering reporting. In the experiment, participants were asked to maintain a 20 m distance from the lead vehicle and to exert lateral control to stay in the lane. The lead vehicle was driven at a speed of 80 km/h and braked at randomly selected times. While participants performed the car-following task, they were also instructed to report their mind wandering state after hearing a brief thought probe tone (duration 0.3s) by pressing buttons on the steering wheel. Participants were told to report “MW-present” or “MW-absent” based on the thoughts immediately preceding the tone. If the thoughts were unrelated to the driving task, such as planning their schedule or “zoning out”, they should report “MW-present”. If the thoughts concerned with maintaining distance from the lead vehicle and keeping in the lane, they should report “MW-absent”. The driving task lasted for 25 min. The first minute allowed the participants adjust the distance from the lead vehicle to the 20m. Starting from the 2nd minute, in every minute, the lead vehicle braked once, and the tone probe occurred once. The tone probe did not occur when the lead vehicle braked, decelerated or accelerated to normal speed, or changed direction in the 5th, 10th, 15th, 20th, and 25th mins.

## Model building

**Fig 2:**
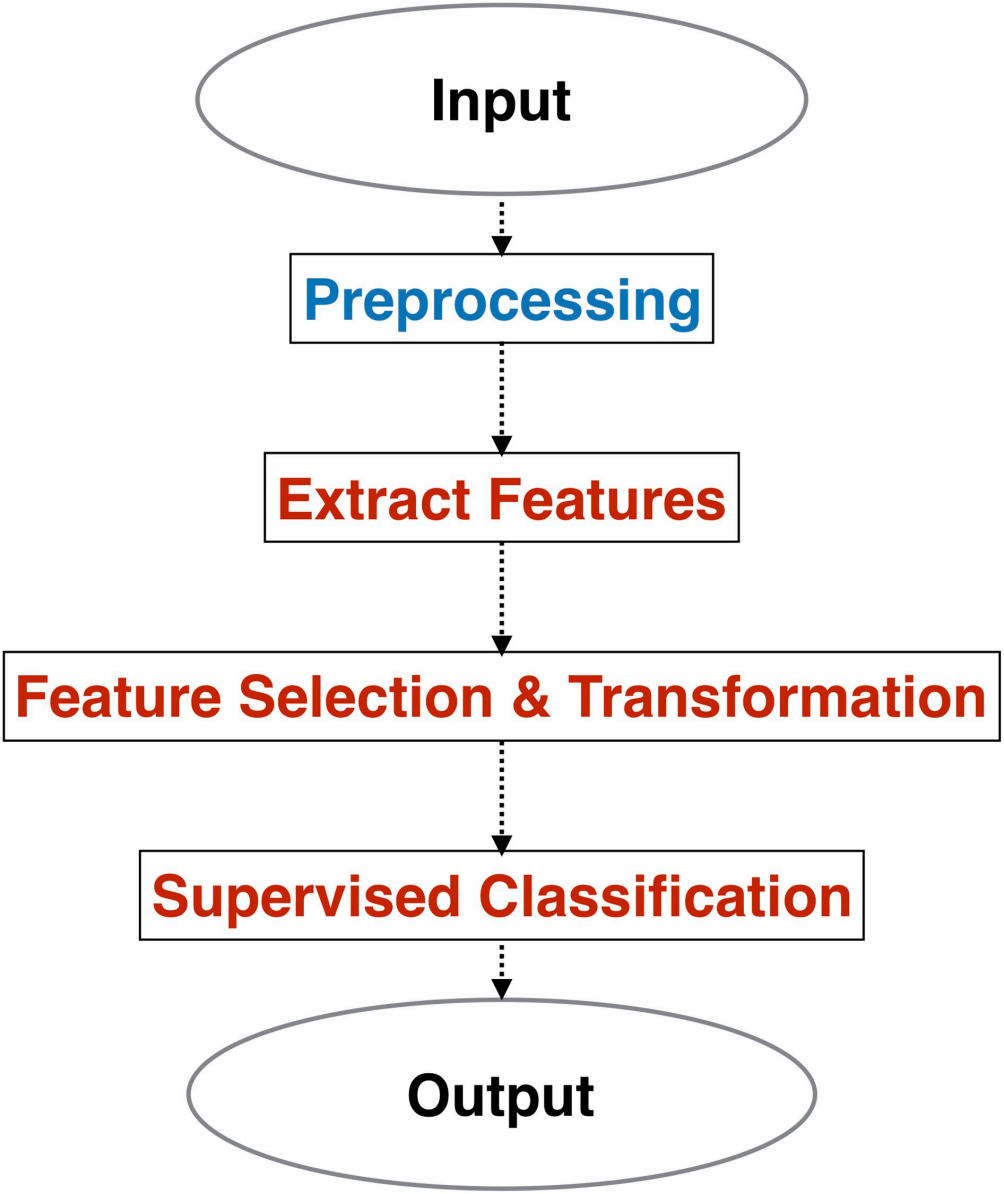
Model building process.

The driving behavior variables are time series. The driving behavior variables time series segment just before tone probe could correspond to the reported mind wandering state. So we got a data set consisting of two pre-labeled classes MW-absent and MW-present. The propose here is to determine the mind wandering state given a segment of driver behavior variables, which is a data mining task—accurately speaking a multivariate time series classification problem. In other words, we need to building model which could distinguish one class from another; then the model can automatically determine to which class of unlabeled dataset belong to. The process to solve such problem is showed in Fig 2.

### Driver-independent modeling

#### Preprocessing

Variables Selection. The main driving task was keeping the car in the lane while maintaining a certain distance from the lead vehicle. Four time series variables related to the driving behaviors of lane-keeping and inter-car distance-keeping were selected from driving log data. Two variables, ‘offset from lane center’ (referred to as ‘offset’) and ‘steering wheel ratio’ (‘steering’), described the participant’s performance on lateral position control; the other two variables, acceleration or deceleration of a car by pushing the foot pedal (‘foot operation’) and ‘distance from the lead vehicle’ (‘distance’), described the performance on longitudinal control. ‘Offset’, with units of meters, was negative if the participant’s vehicle deviated to the left of lane center; the value was positive if the vehicle deviated to the right. ‘Steering’, ranging from -1 to +1, was defined as the steering angle relative to the maximum turn angle. The maximum left turn corresponded to the value of -1 and maximum right turn corresponded to the value of +1. The ‘Steering’ relative to the road curvature would been considered. In the UC-win/Road software, the lead car was set to drive automatically and ideally along the road track, so here the relative ‘Steering’ was calculated by subtracting the lead vehicle’s steering from the participants’ steering at the same position. The values of ‘foot operation’ ranged between -1 and +1, with positive values when the participant pushed the accelerator pedal and negative values when the participant pushed the brake pedal. ‘Distance’, with units of meters, was measured by the distance between the front center of the lead vehicle and the participant’s car.

Data Transformation. To detect mind wandering by building driver-independent models, the four selected variables were preprocessed by three different methods: unmodified data (‘None’), standardized data (‘z-score’) and P-ICA [18]. P-ICA is a simple but efficient ICA (independent component analysis) method, which finds a linear transformation that maximizes the statistical independence between the components of the resulting random vector.

#### Feature type

To build the models, we calculated two types of features: global features and local features. Global features were extracted from the entire driving session of each participant (starting from the 2nd minute), which indicated the different characteristics between individuals. For each preprocessed variable, we calculated four features: maximum value (‘max’), minimum value (‘min’), mean value (‘mean’), and standard deviation (‘std’). Thus each trial of mind wandering reporting had 16 (4 variables × 4 features) global features. The value of global features for each participant were showed in S1 Table. Local features were extracted from the preprocessed variables in the window just before the tone probe for participant’s mind wandering report. For each variable, 21 features were computed in each window: mean value (‘mean’), standard deviation (‘std’), maximum value (‘max’), minimum value (‘min’), median value (‘median’), kurtosis, skewness, intercept, slope, goodness of fit of the linear regression between the variable and time in the window, difference between the end value and the beginning value of the variable (‘diff’), Shannon entropy (‘entropy’), coefficients of a 5-order autoregressive model (AR; 5 features, ‘ar1’, ‘ar2’, ‘ar3’, ‘ar4’, ‘ar5’), and the variance of coefficients for the 3-level 1-D wavelet decomposition (4 features: ‘var(cd1)’, ‘var(cd2)’, ‘var(cd3)’, ‘var(a3)’). Thus there were 84 (4 variables × 21 features) local features for each trial of mind wandering reporting. These local features could show the variability of one participant’s behavior, which could be referred to as ‘intra-individual features’. In previous studies [9, 19, 20], a window size of about 10 seconds directly preceding a probe was employed. Here we calculated local features within three different window lengths: 5s, 10s and 15s.

#### Feature selection and transformation

Before training the classifiers, we applied feature selection to narrow down the number of features in each model. Here we considered firstly using univariate feature selection method, to select features that significantly different between MW-absent class and MW-present class with both two-tailed t-tests and non-parametric Wilcoxon rank sum tests (p < 0.05).

Then the selected features were normalized and transformed by PCA. We tried 6 heuristics—the principal components that explained 50%, 60%, 70%, 80%, 90%, 100% of the total variance were chosen for further analysis [21].

#### Classifier

We built models that discriminated MW-present state from MW-absent state using six supervised machine learning algorithms: support vector machine (SVM), decision tree, ensemble learning, k-nearest neighbor (KNN), discriminant analysis, and Naive Bayes. All classifications were performed using MATLAB (The Mathworks, Inc.). For the ensemble learning parameters, ‘Method’ was set as ‘AdaBoostM1’, ‘NLearn’ was set as ‘100’, and ‘Learners’ were ‘Tree’. The parameters of the other machine learning classification methods were default MATLAB settings.

**Fig 3:**
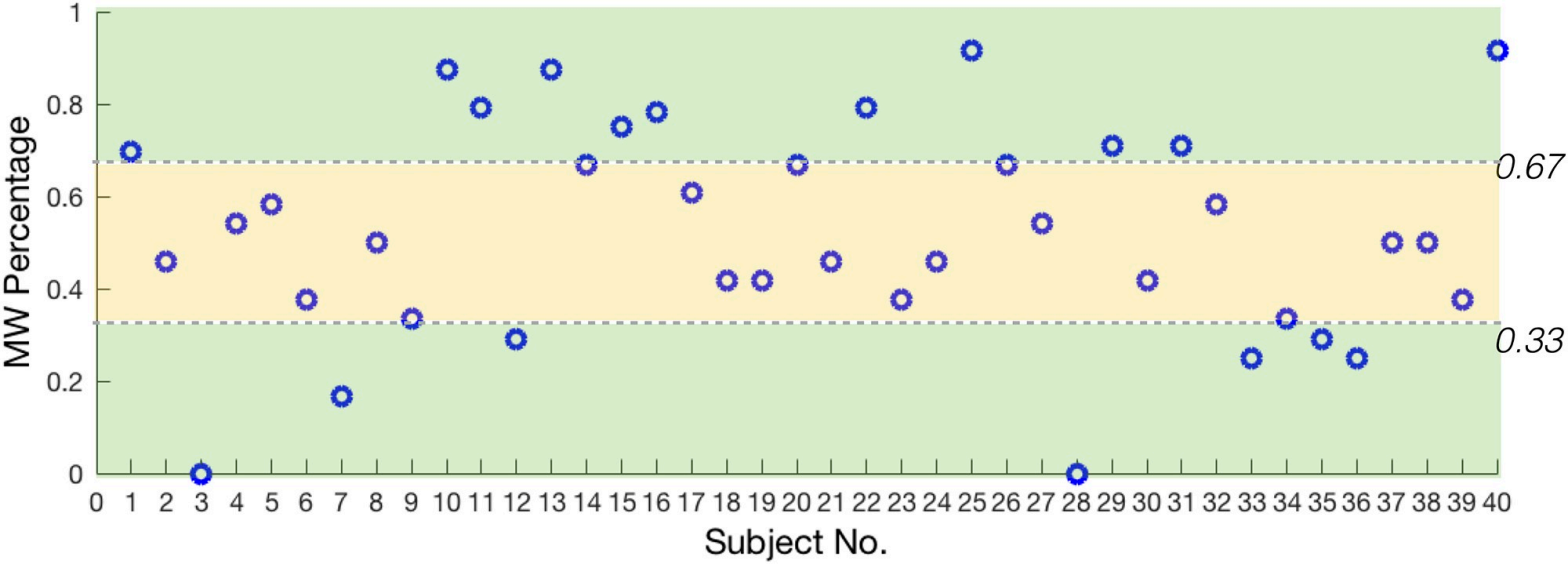
The MW proportion of each participant.

We built models for participants with high or low MW percentage (more than 0.67 or less than 0.33, N = 18) and participants with medium MW percentage (more than 0.33 and less than 0.67, N = 22) separately. The Fig 3 showed the MW proportion of each participant. Global features and local features were entered into the model in three ways: global features only, local features only, and combining global and local features. For the feature type ‘global-only’ we computed all combinations of 3 preprocessing methods × 6 feature selection methods × 6 classifiers. For the feature types ‘local-only’ and ‘combining global and local features’, we performed combinations of 3 preprocessing methods × 3 window sizes × 6 feature selection methods × 6 classifiers.

#### Model validation

To ensure that data from each participant were included in either the training set or the testing set, the leave-one-participant-out cross-validation method was used. This method trained on the data set except one participant and predicted the participant who was not included in the training [4, 7]. Every time one participant was leave out, then performance of the model was averaged across repetitions. To evaluate model performance, we used kappa value [22, 23], accuracy, precision, and recall. The optimal model was the one that produced best kappa values over all combinations.

### Driver-dependent modeling

Because of the individual differences in driving behavior, we also estimated mind wandering in driver-dependent manner, of which each individual was classified directly with local features. We examined three ways to preprocess the selected variables: unmodified data (‘raw’), standardized data (‘z-score’), and P-ICA. We calculated local features with three different window lengths: 5s, 10s and 15s. We selected the top 50% of features ranked by correlation-based feature selection (CFS), which sorts features according to pairwise correlations [24, 25]. After feature selection, PCA was applied. The principal components that explained 50%, 60%, 70%, 80%, 90%, 100% of the total variance were kept. We classified with SVM [26]. The SVM classifier used default settings, in which the default kernel function was ‘linear’. For each participant, we performed combinations of 3 preprocessing methods × 3 window sizes × 6 feature selection methods × 1 classifier. The selected features, preprocessing method, and local features were the same as described above. The 4 time 5 folds cross-validation method was used. Participants who reported extreme proportion of mind wandering occurrences, either very high or very low MW proportion (more than 0.67 or less than 0.33, N = 18) tended to be unbalanced between reported MW-present and MW-absent trials. Thus here when we detected mind wandering using the driver-dependent method, we only built models for participants with medium MW proportion (more than 0.33 and less than 0.67, N = 22).

## Results

There are a total of 40 participants each with 24 thought probes in the driving task. Of the 960 (40 participants × 24 probes) mind wandering thought probes, 3 trials had no reporting. Thus, the dataset used in the classification contained 957 samples, of which 498 samples were labeled with MW-present and 459 samples were labeled with MW-absent. This was close to an equal class distribution.

**Fig 4:**
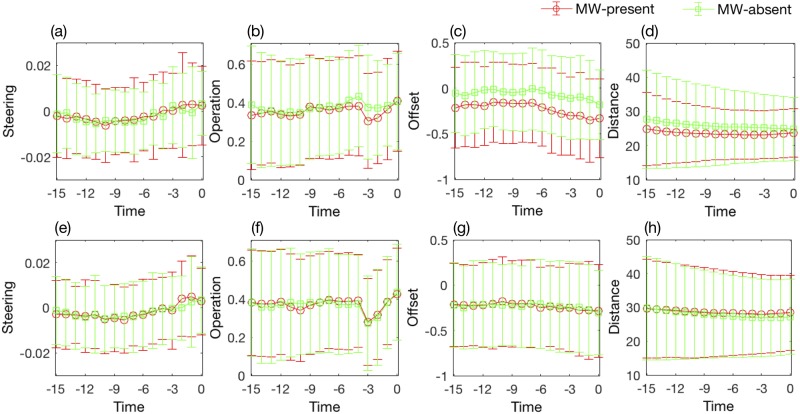
The one standard deviation along the mean of selected variables in the 15s window just before the tone probe calculated across MW-present and MW-absent samples of participants with high or low MW (a, b, c, d), and of participants with medium MW (e, f, g, h). The ‘0s’ on the ‘Time’ axis is corresponding the time of tone probe appearing.

Before building models, we did some basic statistic analysis. The Fig 4 showed the one standard deviation along the mean of selected variables in the 15s window just before the tone probe calculated across MW-present and MW-absent samples of participants with high or low MW (Fig 4a, 4b, 4c and 4d), and of participants with medium MW (Fig 4e, 4f, 4g and 4h). In each subplot, the standard deviation errors bars of MW-present and MW-absent overlapped quite a bit. And the error bar of participants with medium MW overlapped more than participants with high or low MW. It seemed that the ‘offset’ variable would be important to distinguish MW-present and MW-absent state for participant with high or low MW.

The features extracted from the selected variables without transformation (‘None’) that differed significantly between MW-present and MW-absent instances on both t-test and non-parametric Wilcoxon rank sum test (p < 0.05) were calculated. The number of significant global and local features with different windows size (5s, 10s and 15s) for participants with high or low MW and participants with medium MW were showed in Table 1 (S2 Table). For participants with medium MW, there were few significant features.

**Table 1:** The number of significant global and local features with different windows size (5s, 10s and 15s) for participants with high or low MW (‘High&Low’) and participants with medium MW (‘medium’).

	Global	Local, 5s	Local, 10s	Local, 15s
High & Low	13	28	28	30
Medium	0	2	0	2

In addition, the experiment in this study is the same one with the Zhang and Kumada (2017) [17]. The result in the Zhang and Kumada (2017) [17] showed that mind wandering frequency significantly increases over time, and the correlation coefficient between mind wandering frequency and performance measures (standard deviation of lane position (SDLP), standard deviation of steering wheel movements (SDSTW) and standard deviation of foot operation (SDFO)) was significant.

For the driver-independent modeling method, the mind wandering prediction performance of the best models (highest kappa) with global-only, local-only, and both global and local features for participants with high or low MW (Table 2) and for participants with medium MW (Table 3) were presented. For participants with high or low MW, the best model build with global-only features had higher kappa value than models built with other feature types, which had a kappa of 0.384, accuracy of 70%, precision of 71.1%, and recall of 77.9%. For this model, the preprocessing method was z-score, the transformed features explained 80% of the total variance were used, and the classification method was SVM. The predicted accuracy of this model for each participant who had with high or low MW was presented in S1 Table. For participants with medium MW, the optimal model was with local-only features, which had a kappa of 0.124, accuracy of 56.2%, precision of 54.9%, and recall of 58.9%. In this model, the selected variables were preprocessed by z-score, the transformed features explained 50% of the total variance were used, window size was 15s, and KNN was applied as the classification method.

**Table 2:** The best driver-independent model (highest kappa value) built with each feature type for participants with high or low MW and Friedman’s test result.

	Preprocess	Window	Feature Select	ML method	Accuracy	Kappa	Precision	Recall	Friedman’s test
Global	z-score	-	80%	SVM	0.7	0.384 (p < 0.05)	0.711	0.779	*χ*^2^(107) = 126.34 (p > 0.05)
Local	None	5s	100%	Decision Tree	0.624	0.238 (p < 0.05)	0.657	0.668	*χ*^2^(323) = 416.69 (p < 0.05)
Global & Local	z-score	5s	80%	SVM	0.649	0.285 (p < 0.05)	0.680	0.7	*χ*^2^(323) = 299.07 (p > 0.05)

**Table 3:** The best driver-independent model (highest kappa value) built with each feature type for participants with medium MW and Friedman’s test result.

	Preprocess	Window	Feature Select	ML method	Accuracy	Kappa	Precision	Recall	Friedman’s test
Global	P-ICA	-	50%	KNN	0.518	0.021 (p > 0.05)	0.528	0.147	*χ*^2^(107) = 35.67 (p > 0.05)
Local	z-score	15s	50%	KNN	0.562	0.124 (p < 0.05)	0.549	0.589	*χ*^2^(323) = 404.75 (p < 0.05)
Global & Local	z-score	15s	80%	Decision Tree	0.556	0.110 (p < 0.05)	0.552	0.496	*χ*^2^(323) = 268.25 (p > 0.05)

**Fig 5:**
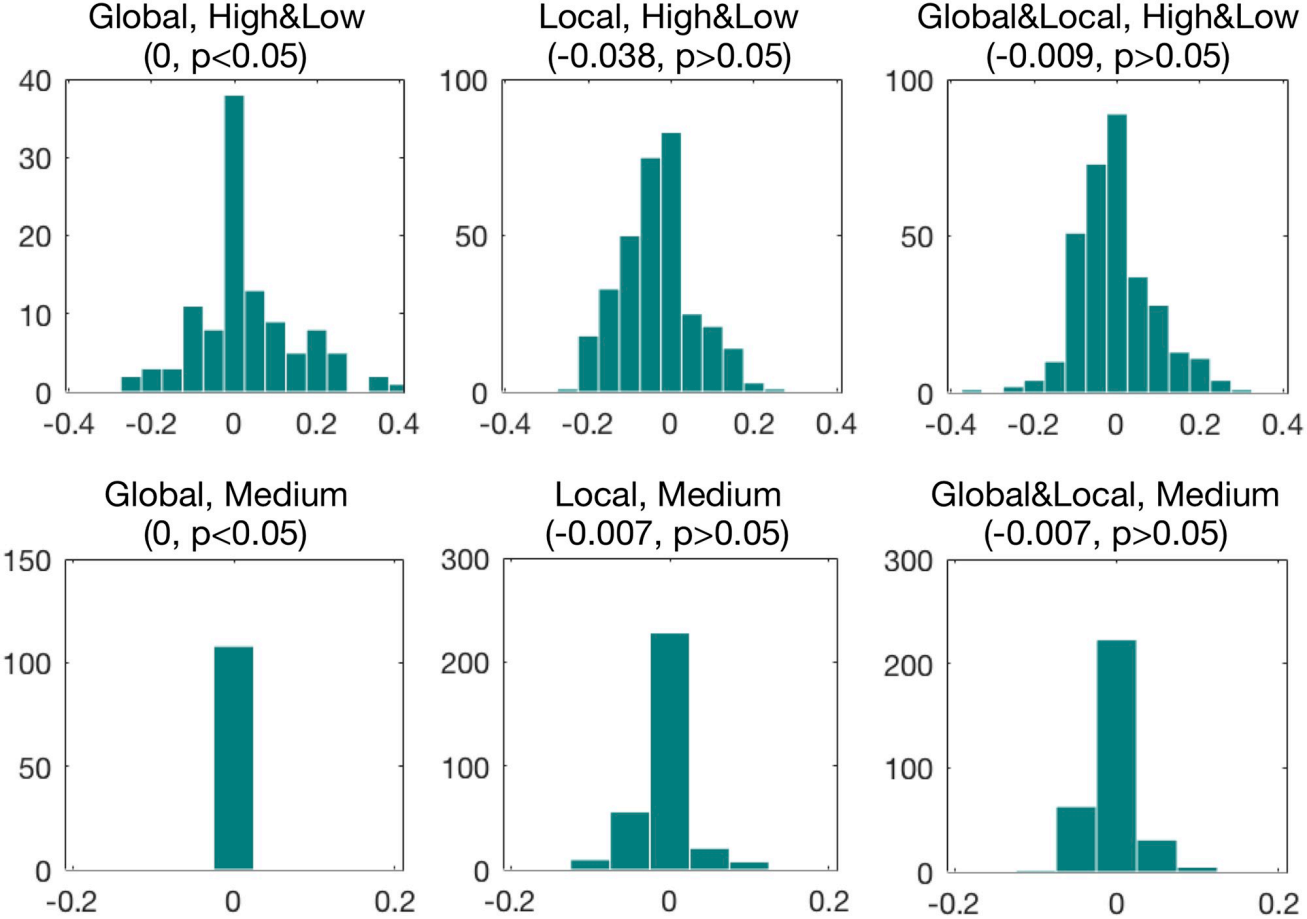
The histogram of the kappa values of the models built with each feature type (‘Global’, ‘Local’, ‘Global&Local’) for participants with high or low MW (‘High&Low’) and for participants with medium MW (‘Medium’). On the top of each subplot, the parentheses included the median kappa value, and the significance of the sign test whether the median kappa value significantly more than 0.

To confirm whether a model offered a significant improvement over other models, the statistical tests (Friedman’s test) was performed on the accuracy values got from each trial of cross-validation folds [27, 28]. The last column of Tables 2 and 3 showed the significance of Friedman’s test. Once Friedman’s test rejects the null hypothesis, we proceeded multiple comparisons in order to find the concrete pairwise comparisons which produce differences [28]. The optimal models demonstrated in Tables 2 and 3 did not have significantly improved prediction performance than other models built with the same feature type. The histogram of the kappa values of the models built with each feature type for participants with high or low MW and for participants with medium MW is showed in Fig 5.

**Fig 6:**
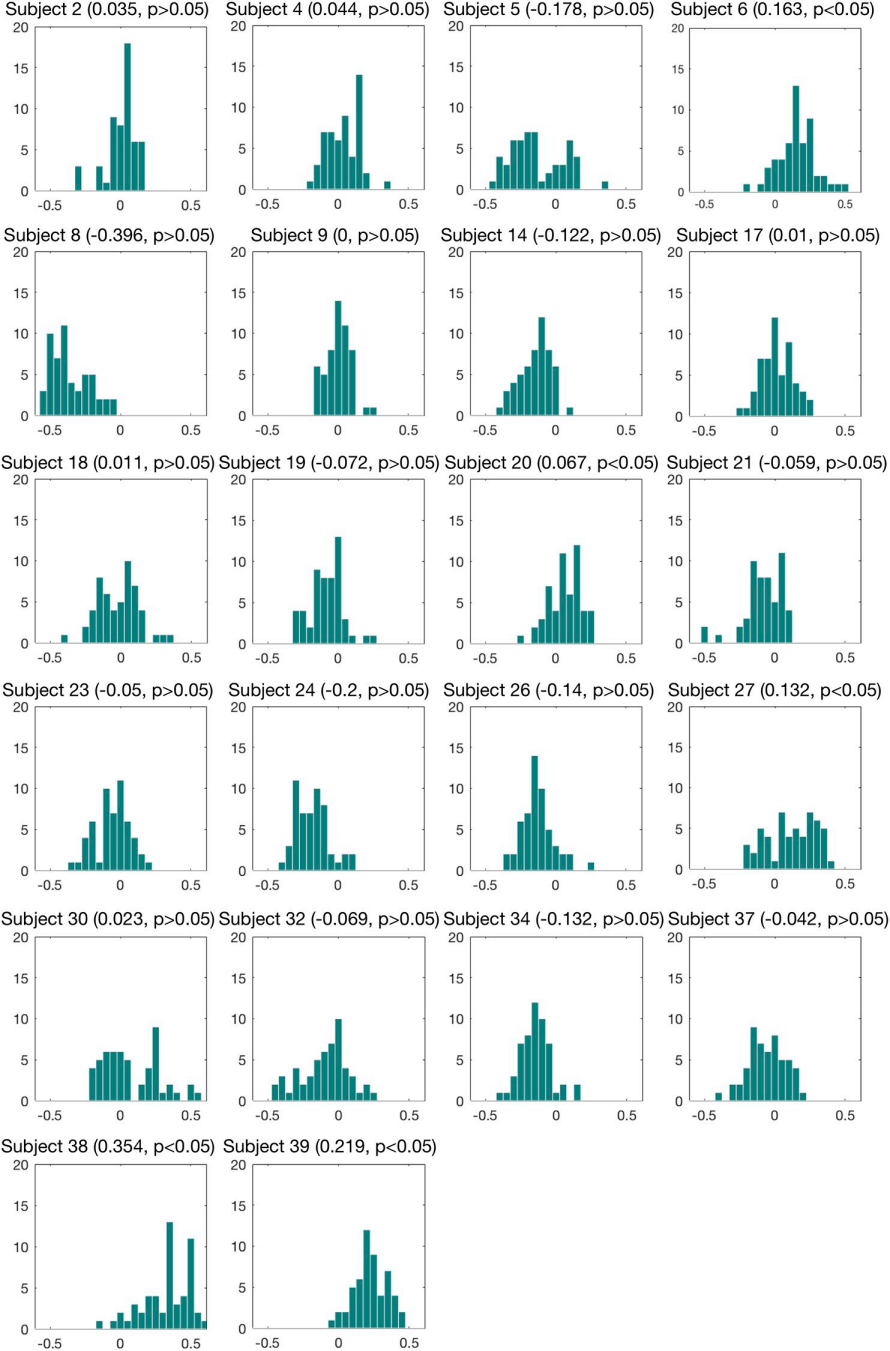
Histograms of the kappa values of the models built for each participant. On the top of each subplot, the parentheses included the median kappa value, and the significance of the sign test whether the median kappa value significantly more than 0.

For the driver-dependent modeling method, each participant with medium MW had 54 model combinations (3 preprocessing methods × 3 window sizes × 6 feature selection methods). The best models (highest kappa value) of each participant were shown in Table 4. We used Friedman’s test to detect significant differences of the models built for each participant. The significance was showed in the last column of Table 4. And we did multiple comparisons test for participants who were significant in Friedman’s test and whose best model had significant kappa value at the same time (Subject 4, 5, 6, 17, 18, 19, 20, 26, 27, 30, 32, 38, and 39). For Subject 17 and 20, there were no significant pairwise models. For Subject 4, 5, 6, 18, 19, 26, 27, 30, 32, 38, and 39, the multiple comparisons test showed that the model with highest kappa value offered a significant improvement than other models. The last row of Table 4 presented the averaged accuracy, kappa value, precision and recall across the best models of each participant. The histograms of the kappa values for each participant are showed in Fig 6. The median kappa values of Subject 6, 20, 27, 38, and 39 were significantly more than 0.

**Table 4:** The best driver-dependent models for participants with medium MW and Friedman’s test result. The subject’ ID with ‘*’ are the subjects whose best model offer a significant improvement than other models.

Subject ID	Preprocess	Window	Feature Select	Accuracy	Kappa	Precision	Recall	Friedman’s test
2	P-ICA	15s	90%	0.594	0.175 (p > 0.05)	0.564	0.500	*χ*^2^(53) = 77.37 (p < 0.05)
4*	P-ICA	10s	100%	0.688	0.373 (p < 0.05)	0.720	0.692	*χ*^2^(53) = 89.09 (p < 0.05)
5*	P-ICA	15s	80%	0.688	0.348 (p < 0.05)	0.717	0.768	*χ*^2^(53) = 172.63 (p < 0.05)
6*	None	10s	100%	0.760	0.486 (p < 0.05)	0.686	0.667	*χ*^2^(53) = 95.18 (p < 0.05)
8	P-ICA	10s	90%	0.479	-0.042 (p > 0.05)	0.478	0.458	*χ*^2^(53) = 150.47 (p < 0.05)
9	P-ICA	10s	100%	0.688	0.274 (p < 0.05)	0.536	0.469	*χ*^2^(53) = 48.45 (p > 0.05)
14	z-score	5s	100%	0.604	0.109 (p > 0.05)	0.703	0.703	*χ*^2^(53) = 176.63 (p < 0.05)
17	P-ICA	10s	80%	0.652	0.263 (p < 0.05)	0.707	0.732	*χ*^2^(53) = 78.27 (p < 0.05)
18*	z-score	5s	90%	0.688	0.343 (p < 0.05)	0.647	0.550	*χ*^2^(53) = 133.77 (p < 0.05)
19*	P-ICA	15s	100%	0.635	0.242 (p < 0.05)	0.568	0.525	*χ*^2^(53) = 87.94 (p < 0.05)
20	None	10s	90%	0.688	0.274 (p < 0.05)	0.750	0.797	*χ*^2^(53) = 74.95 (p < 0.05)
21	z-score	5s	70%	0.563	0.122 (p > 0.05)	0.522	0.545	*χ*^2^(53) = 125.84 (p < 0.05)
23	None	5s	100%	0.635	0.191 (p > 0.05)	0.517	0.417	*χ*^2^(53) = 81.61 (p < 0.05)
24	P-ICA	10s	90%	0.552	0.096 (p > 0.05)	0.512	0.500	*χ*^2^(53) = 87.44 (p < 0.05)
26*	z-score	15s	100%	0.677	0.231 (p < 0.05)	0.732	0.813	*χ*^2^(53) = 146.66 (p < 0.05)
27*	z-score	15s	80%	0.708	0.404 (p < 0.05)	0.700	0.808	*χ*^2^(53) = 171.04 (p < 0.05)
30*	P-ICA	10s	100%	0.792	0.574 (p < 0.05)	0.738	0.775	*χ*^2^(53) = 197.9 (p < 0.05)
32*	None	5s	100%	0.635	0.231 (p < 0.05)	0.667	0.750	*χ*^2^(53) = 161 (p < 0.05)
34	None	15s	100%	0.615	0.153 (p > 0.05)	0.429	0.469	*χ*^2^(53) = 153.83 (p < 0.05)
37	P-ICA	15s	80%	0.604	0.208 (p > 0.05)	0.583	0.729	*χ*^2^(53) = 85.88 (p < 0.05)
38*	P-ICA	10s	80%	0.792	0.583 (p < 0.05)	0.759	0.854	*χ*^2^(53) = 197.73 (p < 0.05)
39*	None	10s	70%	0.760	0.456 (p < 0.05)	0.760	0.528	*χ*^2^(53) = 93.69 (p < 0.05)
Mean Value	-	-	-	0.659	0.277	0.636	0.637	-

## Discussion

Mind wandering is a frequent phenomenon that has a negative impact on performance. The purpose of this study was to detect mind wandering during driving by analyzing driving behavior features. We investigated two types of modeling method, driver-independent and driver-dependent, to identify mind wandering states.

In the driver-independent modeling method, we use the leave-one-participant-out cross-validation method. And we separately build models for participants with high or low MW (Table 2) and participants with medium MW (Table 3), which is similar to the study of Bixler and D’Mello [7]. Bixler and D’Mello [7] considered mind wandering trials at the end and in the midst of reading a page separately.

For participants with high or low MW, we extracted many significant features that significantly different between MW-present and MW-absent samples (Table 1). The participants with high MW or participants with low MW had obvious driving behavior traits. We could distinguish whether the participants have high or low mind wandering percentage with features calculated in a rather large time interval, in our case, which are called ‘global features’ and calculated in a 24 minutes period. Global features were easy to calculate, and possibly compute in a context of computing capability limitations. The optimal model for participants with high or low MW was built with global-only features, and had a significant kappa value of 0.384 (Table 2). The global features contain the information of individual difference. This result indicated that considering individual difference is essential for mind wandering detection. In the multiple comparisons among all models with global-only features, the best model can not significantly better than other models. For participants with medium MW, there were few significant features between MW-present and MW-absent samples (Table 1). And the optimal model was built with local-only features (Table 3), which was not significantly better than other models. So building effective driver-independent models with the leave-one-participant-out cross-validation method is challenging.

In the driver-independent modeling method, the kappa value does not represent moderate agreement between human and automated raters [23]. This was true not only in the reading task of previous studies, but also in our driving task. This may have occurred because objective and subjective measurements tended to reflect different aspects of the mind wandering state [29]. For the subjective reporting, at times, individuals are difficult to categorize a nuanced experience that does not lend to a simplistic distinction between on-task and mind wandering state. And sometimes participants may lack sufficient meta-awareness of their thought content, they will be uncertain to report whether they focus on the task or mind wandering [30].

The executive control is engaged to sustain goal actively and accessibly and to inhibit external distraction and mind wandering. Cognitive and neural resource ability of executive control is individually different and variate intra-individually, such as, sleep deprivation affect executive control [31]. Mind wandering level could also vary not only inter-individually, but also inter-individually. In the driver-dependent classification analysis, the best models for participants with medium MW had an averaged kappa value of 0.277 and accuracy of 65.9% (Table 4). It was better than the driver-independent classifier with local features (kappa value, 0.124; accuracy, 56.2%. Table 3). For Subject 6, 27, 38, and 39, the median kappa values of were significantly more than 0, and the model with highest kappa value offered a significant improvement than other models. So the extracted local features here were rather effective. This result indicates that individual difference could affect prediction performance greatly and building model for each individual could improve the accuracy of mind wandering detection. In this experiment, the collected samples from one participant were small. We anticipated that the driver dependent model could get much better result with enough samples.

Mind wandering detection has relevance to researchers in other fields of psychology. Any human-machine interface would likely benefit from modeling mind wandering [7]. For instance, as driving environment become more complex and dynamic and in-vehicle technologies are more complex, monitoring the attention status is vital for maintaining safe driving. For many psychological studies, such as, memory, visual perception, motor control, and so on, mind wandering is a nuisance and unavoidable variable. Measuring mind wandering with our approach could take into consideration of its confounding effect [29].

Some limitations of our results should be noted. (a) Our experiment was designed on driving simulator, and the driving task and driving environment was simple. Future research should employ more diverse settings in lab environments. And the mind wandering level could be quite different between real and simulated driving. So we are also interested in the experiment on real roads. (b) Our participants were young, with mean age of 22.4 years. Future experiments should recruit a more diverse population, including older people. (c) The driver-dependent classification analysis could obtain best accuracy for participants with medium MW. So it’s possible to collect individual’s daily driving performance data building classification model specifically for each individual. (d) Combining behavior measurements with physiological methods, such as EEG features, may yield enhanced classification accuracy. (e) The content of mind wandering is of great importance in the determination of its impact on driving [32]. It is possible to discriminate different types of mind wandering with respect to content.

## Conclusion

The purpose of this article was to build models capable of automatically detecting mind wandering in the driving task. Our work expanded on previous research by detecting mind wandering through the analysis of driving behavior data from both driver-independent and driver-dependent perspectives. In the driver-independent modeling method, we separately build models for participants with high or low MW and participants with medium MW. For participants with high or low MW, the optimal model was build with global-only features (kappa = 0.384). For participants with medium MW, the optimal model was built with local-only features (kappa = 0.124). But the optimal models can not offer a significant improvement than other models. So building effective driver-independent models with the leave-one-participant-out cross-validation method is challenging. In addition, we could distinguish whether the participants have high or low mind wandering percentage with global features, because the participants with high MW or participants with low MW have obvious driving behavior traits. In the driver-dependent modeling method, we built models for each participant with medium MW. The best models of these participants had an averaged kappa value of 0.277. Subject 6, 27, 38, and 39 had significant the median kappa values more than 0, and the model with highest kappa value of these participants offered a significant improvement than other models. The result here indicates that individual difference could affect prediction performance greatly and building model for each individual could improve the accuracy of mind wandering detection. It is expected that the detecting system could automatically adjust for everyone in anytime.

## Supporting information

S1 Table Global features value and correlation coefficient between each global feature and predicted accuracy. The first column was the subject No. The next 16 column were the value of global features for each participant. The next column was the predicted accuracy of the driver independent model built with global-only features for participants with high or low MW. The last column was the predicted accuracy with best driver dependent model for participants with medium MW. The last two row showed the correlation between each global feature and predicted accuracy for participants with high or low MW and for participants with medium MW. The p-value indicated whether the Pearson linear correlation coefficient is significantly different from zero, and if p-value was significant, the correlation coefficient was displayed.
(XLSX)

S2 Table The mean value, standard deviation and significance of each global feature and local feature with different window size (5s, 10s, 15s) in participants group with high or low MW (‘H&L’) and in participants group with medium MW (‘M’). The name of worksheets included the feature type (‘Global’ / ‘Local’), participants groups (‘H&L’ / ‘M’), and window sizes (‘5s’, ‘10s’, ‘15s’).
(XLSX)
